# A systematic approach for introduction of novel treatments to a chronic patient group: sacubitril-valsartan as a case study

**DOI:** 10.1007/s00228-020-02979-w

**Published:** 2020-08-21

**Authors:** Helena Norberg, Ellinor Bergdahl, Karin Hellström Ängerud, Krister Lindmark

**Affiliations:** 1 Department of Public Health and Clinical Medicine, Umeå University, S-901 87 Umeå, Sweden; 2 Department of Integrative Medical Biology, Umeå University, Umeå, Sweden; 3 Department of Nursing, Umeå University, Umeå, Sweden

**Keywords:** Systematic implementation, Healthcare quality improvement, Chronic disease management, Sacubitril-valsartan

## Abstract

**Purpose:**

To develop a model for systematic introduction and to test the feasibility in a chronic disease population. We also investigated how the approach was received by the patients.

**Methods and results:**

The systematic introduction approach is a seven-step procedure: step 1, define a few main criteria; step 2, primary scan patients with the one or two main criteria using computerized medical records/databases/clinical registries; step 3, identify patients applying the other predefined criteria; step 4, evaluate if any examinations/laboratory test updates are required; step 5, summon identified patients to the clinic with an information letter; step 6, discuss treatment with the patient and prescribe if appropriate; and step 7, follow up on initiated therapy and evaluate the applied process. The model was tested in a case study during introduction of the new drug sacubitril-valsartan in a heart failure population. In total, 76 out of 1924 patients were identified to be eligible for sacubitril-valsartan and summoned to the clinic to discuss treatment. Patient experiences with the approach were investigated in an interview study with general inductive approach using qualitative content analysis. This resulted in three final categories: a good approach, role of the information letter, and trust in care.

**Conclusions:**

The systematic introduction approach ensures that strict criteria are used in the selection process and that a treatment can be implemented in eligible patients within a specified population with limited resources and time. The model was effective in our case study and maintained the patient’s confidence in healthcare.

## Introduction

When novel treatments prove to be more effective than conventional therapies, there is a challenge on how the novel treatment should be introduced. Effective implementation is especially important for patients with chronic diseases who may be treated both in primary care and/or specialist clinics. Patients with chronic diseases depend on their physician’s knowledge and interest in trying the new therapy, and there may be a long passive waiting period, which is referred to as clinical inertia [1]. The common organization of healthcare which requires each physician to be educated with the latest knowledge demands extensive education efforts and usually takes several years before most eligible patients have received the treatment [1–3]. There is also a risk that interested physicians overprescribe newly approved therapies slightly outside of the approved indication owing to intensive marketing and promising study results, which is neither cost-effective nor provides the best quality of care [4].

In order to improve the implementation process of novel treatments, we aimed to develop a model for systematic introduction. Using computerized medical records, local databases and clinical registries to identify eligible patients would potentially maximize cost-effectiveness and assure that strict criteria are applied to identify eligible patients within a hospital district. Using strict objective criteria would also help to reduce structural inconsistencies in treatment. Previous studies have shown that older patients and women often receive less evidence-based therapies and patients with moderate renal impairment are less likely to be prescribed guideline-recommended medications [5–7]. We also wanted to develop a model flexible enough to be able to apply within any discipline or healthcare facility.

We developed a seven-step model, and to test this model in clinical practice, we used the heart failure treatment sacubitril-valsartan. Sacubitril-valsartan is recommended in chronic heart failure patients with reduced ejection fraction who are still symptomatic despite treatment with angiotensin-converting enzyme inhibitors (ACEI) or angiotensin receptor blockers (ARB), beta-blockers, and mineralocorticoid receptor antagonists (MRA) [8, 9]. Heart failure patients are treated in both primary care and specialized heart failure clinics, which makes heart failure an ideal chronic disease to test the systematic approach.

The aim with this study was to develop a model for systematic introduction and to test the feasibility of this model on a new treatment of a chronic disease. We also wanted to investigate how such an approach would be received by the patients.

## Materials and methods

### Model

**Fig. 1:**
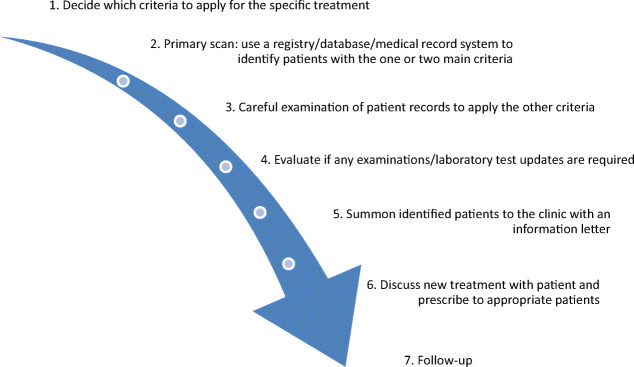
Workflow in the systematic introducing approach

The systematic introducing approach is a procedure of seven steps. Figure 1 summarizes the process:

Step 1: The model starts with defining which criteria to apply for the specific treatment. This requires discussion with hospital administrators responsible for budget allocation. We recommend keeping the criteria as strict as possible to optimize cost-effectiveness, especially when the treatment has not been widespread. Large-scale clinical studies as well as established guidelines should be the foundation of the criteria.

Step 2: Start with the one or two main criteria and perform a primary scan to identify patients. This step is dependent on computerized medical records, databases, or clinical registries.

Step 3: Perform a careful examination of the medical records of the identified patients to apply the other predefined criteria and sort out the patients who have contraindications to the treatment or are obviously not suited for other reasons.

Step 4: Evaluate if any examinations or laboratory test updates are required.

Step 5: Summon the identified patients with an information letter. The letter should contain short information about the new therapy and why they are summoned to the clinic.

Step 6: Discuss the new treatment option with the patient. Explain risks and benefits with the therapy and involve the patient in the treatment decision. Initiate treatment to appropriate patients.

Step 7: The final step is the follow-up with regard to adverse events, dose adjustments, and other aspects depending on the initiated therapy. An evaluation of the process itself should also be performed to evaluate whether or not the predefined criteria were useful in identifying the correct patients or if there would have been an easier way of identifying the patients.

### Study population

#### Case study: implementation of sacubitril-valsartan

Using the suggested model, we attempted a systematic introduction of sacubitril-valsartan in a single-center heart failure clinic in the Umeå University Hospital, Västerbotten, Sweden. The hospital represents the only cardiology clinic in the area, serving approximately 150,000 residents with a mixed rural and urban population. Patients could be included regardless of whether they usually were followed-up in primary or secondary care. The study was performed between April 2016 and January 2018.

In step 1, discussions were held with the local pharmaceutical committee as well as with the head of the cardiology department who is responsible for the budget for cardiovascular pharmaceuticals. The decision was to use the strict entry criteria of the randomized controlled trial, PARADIGM-HF [10], which the approval of sacubitril-valsartan was based upon. Hence, the patients had to fulfill the following criteria: heart failure diagnosis, ejection fraction ≤ 35%, target dose ACEI or ARB, N-terminal pro-B-type natriuretic peptide (NT-proBNP) ≥ 600 ng/L, systolic blood pressure ≥ 95 mmHg, estimated glomerular filtration rate (eGFR) ≥ 30 ml/min, and serum potassium level < 5.4 mmol/L.

In step 2, we used the medical record system of the hospital to identify patients with heart failure and at least one visit at the clinic between January 2010 and March 2016. All patients with an *International Classification of Diseases, 10*^*th*^ *revision* code of heart failure (I50.X, I42.X, I11.0) were identified. The other criteria were then applied one by one.

In step 3, we performed a manual review of the medical records to further exclude patients whose condition had changed or because of other terminal illness were no longer suitable for sacubitril-valsartan.

In step 4, we summoned the patients for an echocardiogram whose latest echocardiography was older than 18 months.

In step 5, identified patients were summoned with an information letter to an outpatient visit. The letter was written and signed by the heart failure cardiologist in charge of the program. A research nurse was responsible for sending the letters.

In step 6, a heart failure cardiologist evaluated other therapies and discussed pros and cons of sacubitril-valsartan with the patient and prescribed the drug if both the physician and patient agreed.

Finally, in step 7, follow-up was performed at 3 and 12 months and an additional 2-week follow-up of blood pressure if systolic blood pressure was ≤ 110 mmHg at baseline visit. At 3 months, the physician called the patients to ask about heart failure symptoms and adverse events. Laboratory tests and blood pressure measurements were repeated from baseline visit. At 12 months, patients were summoned to an outpatient visit where treatment changes were discussed and the routine laboratory tests repeated.

#### Interview study: patients’ experiences

We invited patients to participate in an interview at the baseline visit of the sacubitril-valsartan case study to investigate their perspective of the systematic introduction approach. A total of 24 interviews were conducted with 22 male and 2 female patients. We included patients consecutively, but not all patients were interviewed. No patients declined to participate, but some patients did not have the time to be interviewed before the scheduled baseline visit. All interviews were performed by one member of the research team trained to do semi-structured interviews. If the team member was unavailable at the baseline visit, no interview was conducted. Interviews followed a short interview guide, were audio-recorded, and lasted for 10 to 25 min. For this study, one of the areas from the interview guide was analyzed. This area was about the experience of being summoned to the outpatient visit with an information letter to discuss initiation of a novel drug. The interviews were transcribed verbatim and analyzed using a general inductive approach with qualitative content analysis, inspired by Graneheim and Lundman [11]. Text segments corresponding to the aim of this study were labeled with codes and further sorted into categories. Two members of the research team conducted the coding separately and afterwards discussed coding discrepancies to reach consensus of the final coding.

## Results

### Case study: implementation of sacubitril-valsartan

**Fig. 2:**
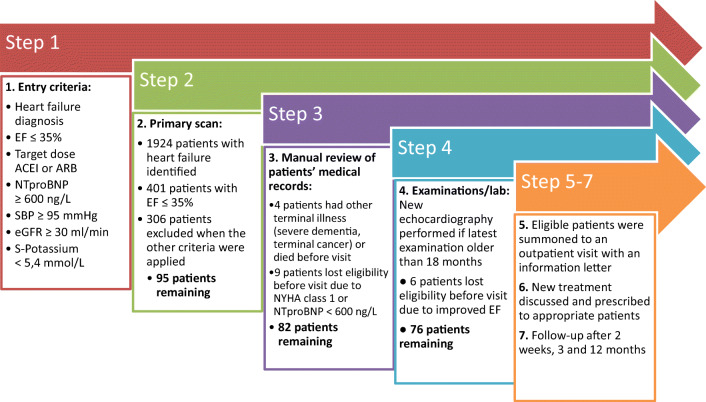
The patient selection process of introducing sacubitril-valsartan to eligible patients. ACEI, angiotensin-converting enzyme inhibitor; ARB, angiotensin receptor blocker; EF, ejection fraction; eGFR, estimated glomerular filtration rate; NT-proBNP, N-terminal pro-B-type natriuretic peptide; NYHA, New York Heart Association; SBP, systolic blood pressure

The selection process of introducing sacubitril-valsartan in a heart failure population is shown in Fig. 2. In step 2, we first identified a total of 1924 patients with a diagnosis of heart failure. After controlling the latest echocardiography, 401 patients had an ejection fraction of ≤ 35%. After applying the rest of the entry criteria, 246 patients did not tolerate target dose ACEI or ARB, 50 patients had NT-proBNP less than 600 ng/L, one patient had eGFR less than 30 ml/min, six patients had systolic blood pressure less than 95 mmHg, and three patients had serum potassium of 5.4 mmol/L or higher. A total of 95 patients remained for step 3.

In step 3, we excluded four patients due to terminal illness (severe dementia, terminal cancer) or death before visit and an additional nine patients who were no longer suitable for sacubitril-valsartan (New York Heart Association Class I, or NT-proBNP less than 600 ng/L).

In step 4, new echocardiography examination was performed in 19 patients, and an additional six patients with improved ejection fraction were excluded. In total, 76 patients fulfilled all predefined criteria. In steps 5 and 6, all 76 patients were summoned to the clinic with an information letter and new treatment discussed. Finally, 70 patients were prescribed sacubitril-valsartan and followed up according to step 7.

### Interview study

In the content analysis, we identified three final categories: a good approach, role of the information letter, and trust in care. Table 1 shows the categories in more detail, also containing identified codes and supportive quotes. In summary, patients were overall satisfied with the new approach and thought it felt reassuring to get an information letter in advance of the outpatient visit. The letter gave them a chance to consider the treatment offer before meeting the physician, although some patients did not understand that they were summoned to discuss a new treatment, despite the information letter. Patients also expressed a trust in care and the follow-up procedure.

**Table 1:** Categories, codes, and exemplar quotes from the interview study

Categories	Codes	Supportive quote(s)
A good approach	It is positive to be summoned	“It feels very good to be summoned.”
	Expecting to be summoned	“It is the service that the citizens are expecting from the hospital, when something new is revolutionary in some way, significantly better than what is now on the market, then you have to either summon or send a letter…”
Role of the information letter	Understanding the letter	“Yes, I got a letter...yes a few weeks ago...a month ago...that they wanted to try a new medicine...and...yes, I'd like to put up so that's why I'm here”
	Feeling reassured by being informed before visit	“Then you were a bit warned.”
	Requesting more information	“I'm interested in percentages, just weighing the two drugs against each other, short-term, long-term on a paper, because I think many people when visit a doctor, you're nervous...then it's a good idea to get an information letter with easy understandable statistics or percentages. Partly before and also afterwards.”
	Not perceiving being summoned for discussion of new treatment	“No...no, it was....it was only that I would take ECG and I'll meet my doctor.”
Trust in healthcare	Feeling reassured with follow-up	“Yes, it [the information letter] said that there was phone contact when needed and after some period it was follow-up, so that...it's nothing they're just sending you home with or what to say...eat this and get well but...they will follow-up some time afterwards....Yes, it feels reassuring, it is nothing you just start with without...”
	Having had good help before	“No, but as I said, I have great confidence in the doctors, I usually say...I have received good help before.”
	Grateful that the healthcare keeping track of me	“Yes, that's because they'll keep track on me...and I'm grateful for that.”

## Discussion

The model of systematic introduction approach was able to identify patients eligible for sacubitril-valsartan in a heart failure population, and the patients were overall satisfied with the approach.

If implementation of new effective treatments is delayed, we cause the patients unnecessary suffering, avoidable hospital admissions, and sometimes even avoidable death as well as creating unnecessary costs. Our case study with sacubitril-valsartan shows that it is possible to introduce a novel therapy to a majority of eligible patients in a specified population with limited efforts and few personnel compared with, e.g., massive education campaigns to every physician that will possibly treat the patients. By using this method, we could also treat patients that otherwise may never had been summoned to the clinic due to being treated in primary care.

**Fig. 3:**
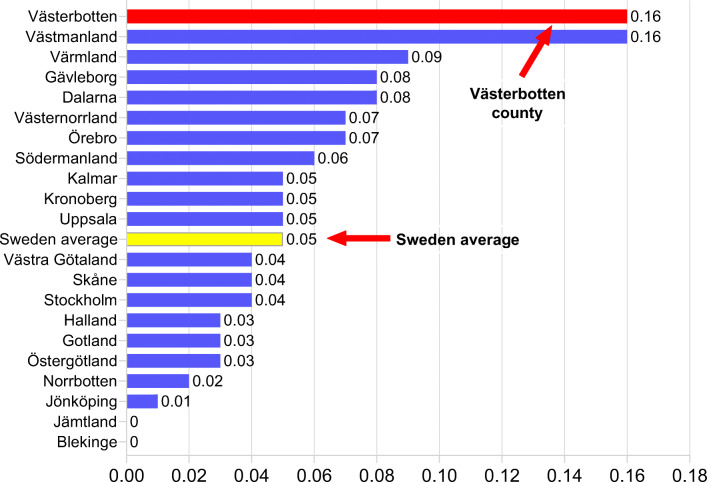
Patients on sacubitril-valsartan/1000 inhabitants in year 2016 [12]

The approach ensures that strict criteria are used in the selection process and thereby helps to optimize cost-effectiveness. The swift and effective introduction of sacubitril-valsartan was reflected in the national drug statistics from 2016 [12], which was the first year after the drug approval. Figure 3 shows that even with our strict interpretation of the PARADIGM-HF study criteria, our county (Västerbotten) had the fastest introduction of sacubitril-valsartan in the country.

Previous studies have shown structural inconsistencies with underrepresentation in clinical trials and less evidence-based therapy in certain patient groups, e.g., women and elderly patients are often underrepresented in cardiovascular trials and receive less diagnostics and drug therapy than males and younger patients [13–17]. We believe that the systematic approach could be used to identify patients on a more objective manner since the model focuses on predefined criteria.

This model is flexible and can be adapted to any discipline and used by any healthcare facility. The crucial factor is to have an electronic record system or registry to identify patients by diagnostic codes or examination results and decide a few main criteria for patient selection. It is important that the criteria are based on robust evidence in order to ensure cost-effectiveness and patient safety. Our case study with heart failure made it necessary to perform step 2 of our model in several stages. As heart failure is a common disease, identifying patients with heart failure and a low ejection fraction was not enough to limit the number of patients eligible for step 3, but with predefined criteria, the number of medical records needing a more detailed review was limited. We believe that a key to this method is to limit the number of patients needing full scrutiny by having easy-to-follow criteria in the selection in order to allow for nurses or junior doctors to facilitate the process.

The model could be efficient also for older drugs or devices, e.g., when underuse of a treatment is suspected in a population. By specifying the criteria, it does not matter if the treatment is newly approved or has been on the market for decades as long as the evidence is still valid.

The interview study showed that the patients were overall positive to this approach. The interviewed patients did in general not question the physician´s suggestion and had confidence in the expertise of the healthcare staff. Interestingly, some patients even had an expectation of being contacted by the healthcare when a new therapy might be appropriate for them. This supports our results of patients having trust in provided healthcare and indicates that the patients are already on-board with this method. They also expressed that the information letter gave them a chance to consider the treatment in advance.

### Limitations

The single-center case study design reduces the generalizability of the results to other regions and disciplines. On the other hand, the population size or discipline is not crucial as long as electronic records or registries are available for the patient selection. Additionally, results from the qualitative study may not be simply extrapolated to other hospitals or treatments as the expectations from the patients and trust in the healthcare system may vary between both patient groups and geographic areas. Some patients did not understand the reason why they were summoned to the clinic, indicating the importance of a clear information letter.

Another limitation is that the approach involves an initial workload. Not just to determine selection criteria and taking time to identify the patients but we found that a sudden influx of patients summoned to the clinic stressed the resources of the clinic.

If the systematic approach is to be used in a larger scale, the workload of the selection process needs to be further reduced. More specific registries that can automatically sort patients according to more than one entry criteria would be ideal.

## Conclusions

We developed a model for introduction of novel therapies for a chronic patient group and tested the model on sacubitril-valsartan in heart failure. The model ensures that strict criteria are used in the selection process and that a treatment can be implemented in eligible patients within a specified population with limited resources and time. The model was effective in our case study and maintained the patient’s confidence in healthcare.

## Data Availability

The datasets used and/or analyzed during the current study are available from the corresponding author on a reasonable request.
